# Patient activation in advanced chronic kidney disease: a cross-sectional study

**DOI:** 10.1007/s40620-023-01847-x

**Published:** 2024-02-12

**Authors:** Laura E. Lunardi, Richard K. Le Leu, Lisa A. Matricciani, Qunyan Xu, Anne Britton, Shilpanjali Jesudason, Paul N. Bennett

**Affiliations:** 1 Central Northern Adelaide Renal and Transplantation Service, South Australia, Australia; 2 Clinical & Health Sciences, University of South Australia, South Australia, Australia; 3 Faculty of Health and Medical Sciences, University of Adelaide, South Australia, Australia; 4 School of Nursing and Midwifery, Griffith University, Queensland, Australia

**Keywords:** Chronic kidney disease, Self-management, Patient activation, Medication adherence

## Abstract

**Background:**

Patient activation refers to the knowledge, confidence and skills required for the management of chronic disease and is antecedent to self-management. Greater self-management in chronic kidney disease (CKD) results in improved patient experience and patient outcomes.

**Aim:**

To examine patient activation levels in people with CKD stage 5 pre-dialysis and determine associations with sociodemographic characteristics, treatment adherence and healthcare utilisation.

**Methods/design:**

People with CKD stage 5 not receiving dialysis from one Australian kidney care service. Patient activation was measured using the 13-item Patient Activation Measure (PAM-13). Sociodemographic and clinical outcome data (emergency department visits, admissions) were collected from medical records. Morisky Medication Adherence Scale was used to determine self-report medication adherence.

**Results:**

Two hundred and four participants completed the study. The mean PAM-13 score was 53.4 (SD 13.8), with 73% reporting low activation levels (1 and 2). Patient activation scores significantly decreased with increased age (*P* < 0.001) and significantly increased with higher educational levels (*P* < 0.001). Higher patient activation level was associated with fewer hospital emergency department visits (*P* = 0.03) and increased medication adherence (*P* < 0.001).

**Conclusion:**

Patient activation levels are low in people with CKD stage 5 not receiving dialysis suggesting limited ability for self-management and capacity for optimally informed decisions about their healthcare. Efforts to improve patient activation need to consider age and education level.

**Graphical abstract:**

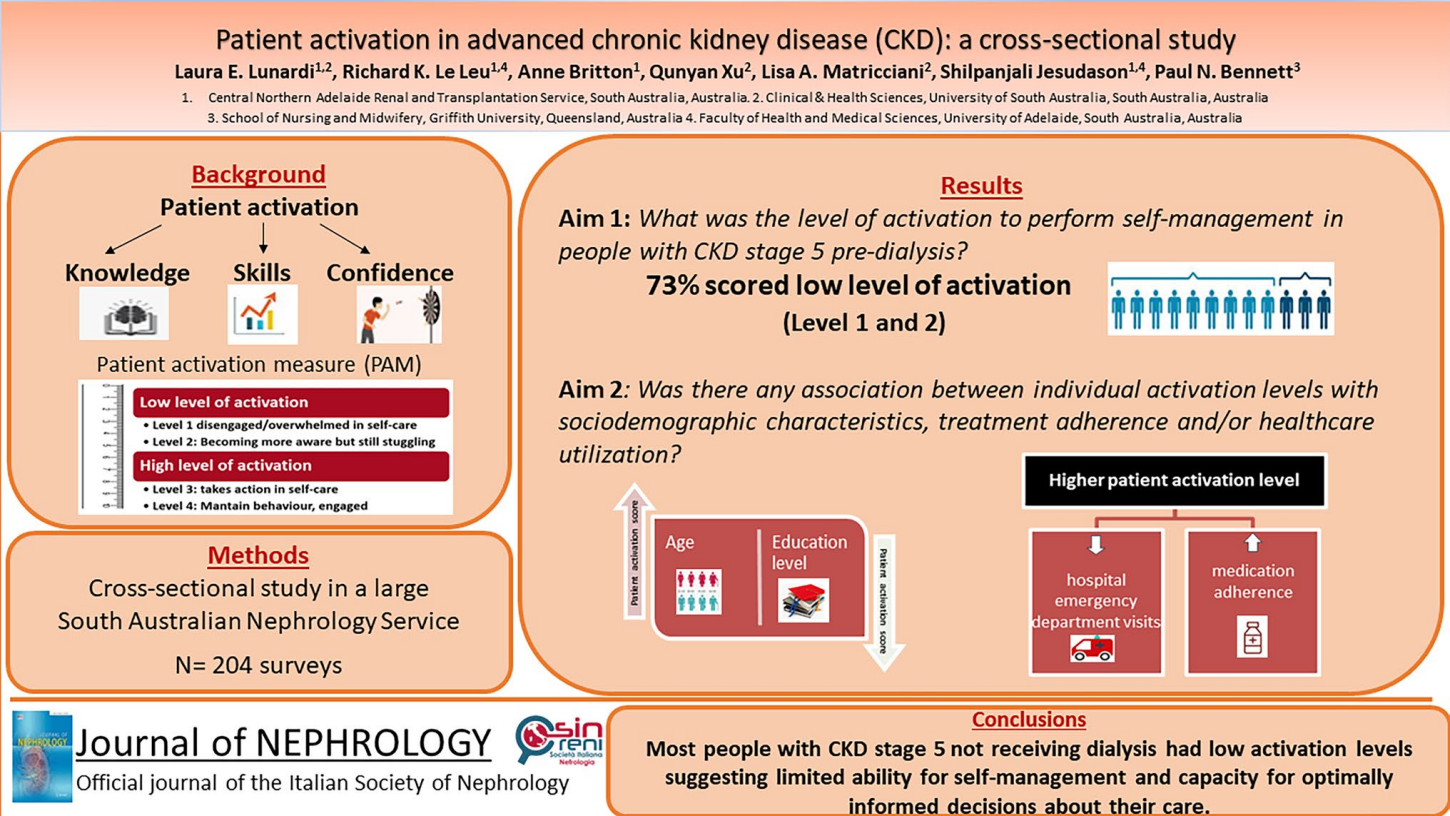

**Supplementary Information:**

The online version contains supplementary material available at 10.1007/s40620-023-01847-x.

## Introduction

Patient activation refers to the skills, knowledge and confidence that contribute to the willingness and ability of a person to manage their own health [1]. Individuals with higher levels of patient activation are more likely to participate in self-management behaviours, have fewer unmet medical care needs and are more likely to access support from health professionals [1, 2]. Increased activation and self-management can improve blood glucose control, diet, exercise, smoking cessation, and weight management [3, 4]. This ultimately reduces unnecessary healthcare utilisation costs and adverse health outcomes that often arise when patients do not effectively engage in their self-management to adopt lifestyle changes recommended by medical, nursing and allied health providers [5, 6]. Conversely, individuals with lower levels of activation are more likely to be hospitalised, have poorer health care experiences, lower adherence to treatment and greater health costs [7–9].

The relationship between patient activation and improvements in health outcomes has been established in chronic conditions such as diabetes [10–12], heart failure [11], chronic obstructive pulmonary disease [11] and kidney failure, including patients undergoing haemodialysis [4, 13, 14]. In the dialysis population, patient activation has been added as a quality metric by the US Centers for Medicare and Medicaid Services (CMS) in the Kidney Care Choices model [15].

Little is known about patient activation in the stage 5 chronic kidney disease (CKD) pre-dialysis population. Chronic kidney disease refers to the progressive reduction of kidney function for a period longer than three months. Chronic kidney disease is categorised into five stages with stage 5 being kidney failure defined by an eGFR of < 15 mL/min/1.73 m^2^ [16]. When patients reach kidney failure they require renal replacement therapy (dialysis or kidney transplant) to sustain life [17].

Patient activation is especially relevant for people with stage 5 CKD not yet receiving dialysis (CKD-ND), as they require the appropriate skills and knowledge to manage their health and face the choice of kidney treatment modalities (hemodialysis, peritoneal dialysis, kidney transplantation or conservative management). Identifying the activation level of individuals with stage 5 CKD-ND could improve care by focusing efforts and resources and guiding interventions. Lower activated individuals are less likely to recognise early warning signs of health complications or organ deterioration, are less motivated to act, and unsure of how to respond to health changes [18]. Therefore, in this study we aimed to investigate the patient activation level in the stage 5 CKD-ND population and explore if there was any association of patient activation level, treatment adherence and healthcare utilisation with sociodemographic characteristics.

## Methods

### Design and study population

A cross-sectional study was conducted on a sample of adults (≥ 18 years of age) with stage 5 CKD-ND The kidney care program (KCP) is composed of two senior CKD nurses and three nephrology nurse practitioners working collaboratively with nephrologists and allied health to provide a single point of contact for patients with stages 4–5 supporting decision-making through information about the different kidney treatment modalities. The kidney care program at the time of this study included 350 patients.

Patients were invited to take part in this study during a six-month period between July and December 2022. Adults with a diagnosis of stage 5 CKD-ND (defined as an estimated glomerular filtration rate ≤ 15 ml/min/1.73 m^2^) were invited via phone, email or face-to-face during clinic attendance. Patients were excluded if they were admitted as an inpatient in hospital, had severe cognitive impairment and/or inability to complete the surveys written in English. Study participants completed the 13-item Patient Activation Measure (PAM-13) and 8-item Morisky Medication Adherence Scale (MMAS-8) questionnaires. Sociodemographic and clinical data were extracted from participant’s electronic medical records and entered into Research Electronic Data Capture (REDCap) software version 1 21.09.2022 [19].

This project received ethical approval from Central Adelaide Health Local Network (CALHN) (reference number 16067), Northern Adelaide Health Local Network (NALHN) (SSA reference number 22-040) and University of South Australia (application ID: 205151) Human Research Ethics Committee and Research Governance. The study was registered with the Australian New Zealand Clinical Trials Registry (ANZCRT) #12622000451707. The PAM-13 instrument is protected by Insignia Health license #1654066536–1685602536. This study complies with the STROBE Checklist for observational cross-sectional studies [20] (Supplemental Table 1).

### Study measures

Patient activation was evaluated using the PAM-13 with a 5 point Likert scale (strongly disagree to strongly agree). A fifth, not applicable (NA) response, was also offered. The scores for this tool were then converted to an overall activation score between 0 and 100, with higher values reflecting higher activation. Levels are designated by cut-off points based on the 2013 PAM license scoring rules (Insignia^®^) [21] and used by Hussein et al*.* [18]*.* PAM-13 scores were categorised into four levels. Level 1 (0.0–47.0) reflects low activation and suggests that the person does not yet understand their role in healthcare; Level 2 (47.1–55.1) indicates the person does not yet have the knowledge and confidence to take action; Level 3 (55.2–72.4) indicates the person is beginning to engage in positive health behaviours; Level 4 (72.5–100) reflects high activation and suggests the person is proactive and engaged in recommended health behaviours [18, 21].

Sociodemographic characteristics operationalised as categorical variables: age (younger than 64 years vs older than 65 years [older adults], classification based on the Australian Bureau of Statistics [22], sex (female vs male), marital status (considered in terms of single [never married], partnered [married or de facto relationship] and separated [divorced or widowed]), current living situation (people living alone, living with family [partner, children or parents] or others [supported by carers or friends]), educational level (below high school [did not finish primary school, did not go to school, did not complete high school], completed high school and higher than high school [college, university degree or postgraduate degree]), and ethnicity (assessed using the Australian Bureau of Statistics (ABS) [23]. Socioeconomic status was calculated using the Socio-Economic Indexes for Areas (SEIFA) Australian Bureau of Statistics [24]. The SEIFA disadvantage score, a quintile based on residential postcode data from the Australian Bureau of Statistics census, classified the postcodes following the Index of Relative Social Disadvantage (IRSD). This IRSD index summarises the socio-economic conditions of people living in an area. Each postcode of the IRSD was categorised into five quantiles: the first quantile represents the most disadvantaged and the fifth quantile represents the least disadvantaged population.

Treatment adherence measures were the number of missed appointments at kidney care and nephrologist clinic in the previous 12 months, and Morisky Medication Adherence Scale (MMAS-8) [25, 26]. The MMAS-8 consists of seven items with the binary response and one item with the Likert scale response. Cumulative scores based on eight items were used to obtain a final adherence score ranging from 0 to 8. Adherence was defined accordingly as low (score 0–5), medium (score 6–7) and high (score 8). MMAS-8 has been validated in studies with good reliability and predictive value [25].

Healthcare utilisation was measured by the number of hospital emergency department visits over the past 12 months and the number of hospital admissions over the prior 12 months.

### Statistical analysis

All patients with stage 5 CKD in the kidney care program were identified and listed in alphabetic order by patient’s last name. Individual patient eligibility was assessed, and those eligible were selected sequentially from the list, assuming simple random sampling has occurred. The overall proportion of each patient activation level in the target population, patients in the kidney care program, was estimated. Sub-sample estimation of each patient activation level, where a sub-sample was defined by the characteristics concerning a sociodemographic factor was also conducted. The strength of evidence for the association between each sociodemographic factor and patient activation level was tested in a modified chi-squared test for contingency table, which considered survey weights. Due to ethics restrictions, no information was collected for non-respondents, hence only crude non-response rate was considered in the post-estimation weight construction.

The relationship between patient activation, treatment adherence and healthcare utilisation variables were examined via regression method after adjusting for confounders. Confounders were informed by expert clinical content knowledge in CKD (LL, PB, SJ) and existing literature [27]. Age and education level were confounders for hospital emergency department visits, hospital admissions and missed renal appointments over the last 12 months. Age, education level, and participant’s home status (self/family/others) were confounders for medication adherence.

A model-based approach, as opposed to a design-based approach, was used in the analysis to allow the findings to be generalised to CKD populations in other settings. Negative binomial distribution was used for count outcome measures, hospital admissions, emergency visits, and missed renal appointments, to account for the overdispersion. Linear regression was used for outcome measure medication adherence score. In modelling each outcome measure, different functional forms of patient activation score were fitted, and the selection of the model was guided by the information criteria and the ease of interpretability of the functional form itself. Statistical significance was indicated by a *P* value of < 0.05.

## Results

### Participant characteristics

**Fig. 1:**
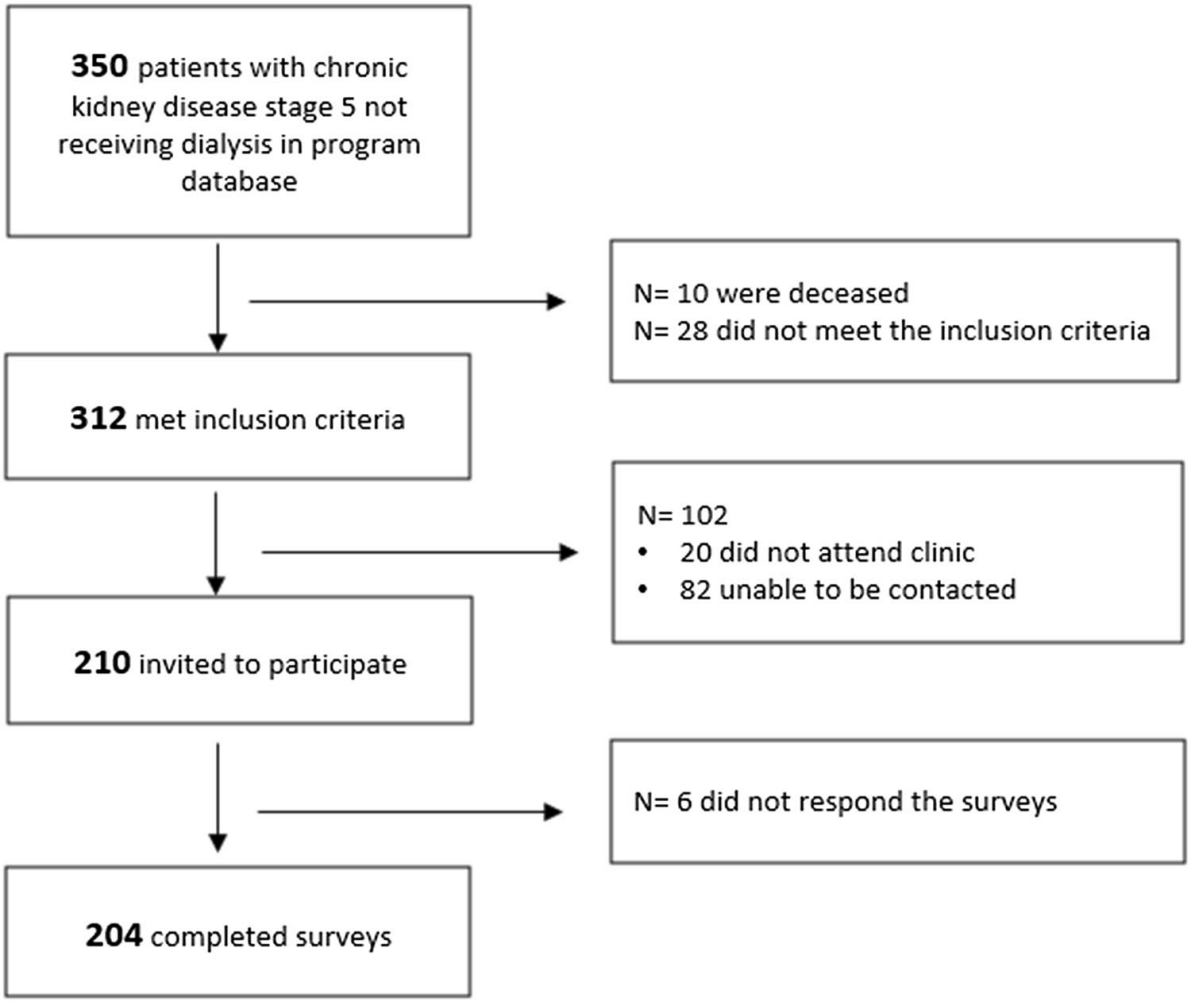
Participant flow chart

In the kidney care program, the total number of patients who met the eligibility criteria was 312. Of these, 210 patients were invited to participate and 6 of them did not respond, resulting in a response rate of 97.1% (Fig. 1). Most respondents were male (61.3%) and were aged between 27 to 91 years, with a mean age of 69 (SD 13.2). Seventy-eight percent self-reported Australian ethnicity, followed by 6% for First Nation People and 5% for Italian. The remaining 11% ethnic groups included Asian (Philippines, Vietnam, China, Korea, India), European (England, Ireland, Croatia). Sixty-three percent reported living with a family (partner/children/parents). Most participants had completed high school education (44%) with 55% of the 204 having lower socio-economic status (1st and 2nd quintile). The mean MMAS-8 score was 6.4 (SD 1.7), indicating a high level of medication adherence (Table 1).

**Table 1:** Demographic characteristics, treatment adherence, health service utilisation and patient activation among people with CKD stage 5 not receiving dialysis (*N* = 204^a^)

Characteristics	Frequency (%) (%)	Mean (SD)	Median (IQR)	Available *N*
Age (years)		69 (13.2)	73 (62—78)	204
Age (years)				
< 65	67 (32.8)			
≥ 65	137 (67.2)			
Sex				204
Male	125 (61.3)			
Female	79 (38.7)			
Education				204
< High school	58 (28.4)			
High school	90 (44.1)			
> High school	56 (27.5)			
Socio-economic status				204
Quantile 1	85 (41.7)			
Quantile 2	28 (13.7)			
Quantile 3	42 (20.6)			
Quantile 4	37 (18.1)			
Quantile 5	12 (5.9)			
Marital status				198
Separated	44 (22.2)			
Partnered	115 (58.1)			
Single	39 (19.7)			
Ethnicity				197
First Nation People	12 (6.1)			
Australian	153 (78.1)			
Other	32 (15.8)			
Persons living with				201
By self	62 (30.8)			
Others	12 (6.0)			
Family (partner/children/parents)	127 (63.2)			
Missed renal appointments^b^				203
0	114 (56.2)			
1–3	84 (41.4)			
4 or more	5 (2.5)			
Emergency visits^b^				204
0	109 (53.4)			
1–4	88 (43.1)			
5 or more	7 (3.4)			
Hospital admissions^b^				202
0	112 (55.4)			
1–4	76 (37.6)			
5 or more	14 (6.9)			
MMAS score		6.4 (1.7)	7 (5.5–8)	204
Patient activation score		54 (13.9)	51 (45.3–56.2)	204
Patient activation				204
Level 1	78 (38.2)			
Level 2	70 (34.3)			
Level 3	32 (15.7)			
Level 4	24 (11.8)			

MMAS = Morisky Medication Adherence Scale. The MMAS-8 Scale, content, name, and trademarks are protected by US copyright and trademark laws. Permission for use of the scale and its coding is required. A license agreement is availablefrom MMAR, LLC., Donald E. Morisky, ScD, ScM, MSPH, http://ww.moriskyscale.com

*IQR* interquartile range, *SD* standard deviation

^a^Total number of observations is *N* = 204

^b^The number of missed renal appointments, emergency visits and hospital admissions were counted over the last 12 months

### Patient activation and sociodemographic characteristics

Overall and sub-sample proportion of PAM scores are shown in Table 2. The mean PAM score was 53.4 (SD 13.8) with the majority of participants 148/204 (73%) scoring a low level of patient activation, either level 1 or level 2. Only 32/204 (16%) participants scored patient activation level 3 and 24/204 (12%) scored level 4. Significantly lower PAM scores were observed among older participants (*P* < 0.001) and those with a lower level of education (*P* < 0.001).

**Table 2:** Estimated overall and sub-sample proportion and 95%CI of each patient activation level (N = 204*) among people with CKD stage 5 not receiving dialysis

	Available N	Estimated proportion (95% CI)				*P* value
		PA level 1	PA level 2	PA level 3	PA level 4	
Overall	204	0.38 ( 0.34, 0.42)	0.34 ( 0.31, 0.38)	0.16 ( 0.13, 0.19)	0.12 ( 0.09, 0.15)	
Age	204					< 0.001
< 65 years		0.28 ( 0.22, 0.35)	0.28 ( 0.22, 0.35)	0.24 ( 0.18, 0.31)	0.19 ( 0.14, 0.26)	
≥ 65 years		0.43 ( 0.38, 0.48)	0.37 ( 0.33, 0.42)	0.12 ( 0.09, 0.15)	0.08 ( 0.06, 0.11)	
Sex	204					0.666
Female		0.41 ( 0.34, 0.47)	0.32 ( 0.26, 0.38)	0.15 ( 0.11, 0.21)	0.13 ( 0.09, 0.18)	
Male		0.37 ( 0.32, 0.42)	0.36 ( 0.31, 0.41)	0.16 ( 0.13, 0.20)	0.11 ( 0.08, 0.15)	
Marital status	198					0.060
Separated		0.45 ( 0.37, 0.54)	0.27 ( 0.20, 0.36)	0.11 ( 0.07, 0.18)	0.16 ( 0.10, 0.24)	
Partnered		0.34 ( 0.29, 0.39)	0.37 ( 0.32, 0.43)	0.17 ( 0.14, 0.22)	0.11 ( 0.08, 0.15)	
Single		0.38 ( 0.30, 0.48)	0.36 ( 0.27, 0.45)	0.18 ( 0.12, 0.26)	0.08 ( 0.04, 0.15)	
Education	204					< 0.001
< High school		0.60 ( 0.53, 0.68)	0.28 ( 0.21, 0.35)	0.10 ( 0.06, 0.16)	0.02 ( 0.01, 0.05)	
High school		0.30 ( 0.24, 0.38)	0.30 ( 0.24, 0.38)	0.18 ( 0.13, 0.25)	0.21 ( 0.16, 0.29)	
> High school		0.29 ( 0.24, 0.35)	0.41 ( 0.35, 0.47)	0.18 ( 0.14, 0.23)	0.12 ( 0.09, 0.17)	
Socio-economic status	204					< .001
Quantile 1		0.46 ( 0.40, 0.52)	0.27 ( 0.22, 0.33)	0.19 ( 0.14, 0.24)	0.08 ( 0.05, 0.12)	
Quantile 2		0.36 ( 0.26, 0.47)	0.5 ( 0.39, 0.61)	0.07 ( 0.03, 0.16)	0.07 ( 0.03, 0.16)	
Quantile 3		0.26 ( 0.19, 0.35)	0.36 ( 0.27, 0.45)	0.14 ( 0.09, 0.22)	0.24 ( 0.17, 0.32)	
Quantile 4		0.41 ( 0.31, 0.50)	0.30 ( 0.22, 0.39)	0.19 ( 0.12, 0.28)	0.11 ( 0.06, 0.19)	
Quantile 5		0.25 ( 0.12, 0.44)	0.58 ( 0.4, 0.75)	0.08 ( 0.02, 0.26)	0.08 ( 0.02, 0.26)	
Persons living with	201					< 0.001
By self		0.32 ( 0.26, 0.40)	0.35 ( 0.29, 0.43)	0.15 ( 0.10, 0.21)	0.18 ( 0.13, 0.24)	
Family		0.37 ( 0.32, 0.42)	0.35 ( 0.31, 0.41)	0.17 ( 0.14, 0.22)	0.10 ( 0.07, 0.14)	
Others		0.75 ( 0.56, 0.88)	0.17 ( 0.07, 0.35)	0.08 ( 0.02, 0.26)	–	

– estimation not considered due to lack of observation in the sample

*CI* confidence interval, *PA* patient activation

### PAM-13 and health service utilisation

Patient activation was associated with the number of hospital emergency department visits (*P* = 0.032) over the last 12 months after adjusting for age and education level. In patients with the same age and education level, the emergency department visits in the last 12 months in patient activation level 3 was 33% of those with a patient activation level 1 (95% CI 0.16–0.70), *P* = 0.012 (Table 3). The number of hospital admissions in the last 12 months in participants with patient activation level 3 was 36% of those with a patient activation level of 1 (95% CI 0.16–0.77, *P* = 0.026) (Table 3), showing no statistically significant association between patient activation level and hospital admissions in this study.

**Table 3:** Association between patient activation with treatment adherence and health service utilisation among people with CKD stage 5 not receiving dialysis

Outcome measure (available *N*)	Patient activation	Crude IRR (95% CI)/coefficient (95% CI)	*P* value	Adjusted IRR (95% CI)/coefficient (95% CI)	*P* value
Emergency department visits^a,c^ (*N* = 203)			0.14		0.032
	Level 1	Ref		Ref	
	Level 2	0.76 (0.44, 1.29)	0.640*	0.66 (0.39, 1.12)	0.296*
	Level 3	0.41 (0.20, 0.88)	0.057*	0.33 (0.16, 0.70)	0.012*
	Level 4	0.83 (0.39, 1.85)	0.949*	0.68 (0.32, 1.50)	0.689*
Hospital admissions^a,c^ (*N* = 202)			0.10		0.061
	Level 1	Ref		Ref	
	Level 2	0.79 (0.47, 1.34)	0.746*	0.76 (0.45, 1.29)	0.642*
	Level 3	0.41 (0.19, 0.87)	0.055*	0.36 (0.16, 0.77)	0.026*
	Level 4	0.58 (0.26, 1.31)	0.431*	0.57 (0.25, 1.30)	0.443*
Missed renal appointments^a,c^ (*N* = 203)			0.056		0.062
	Level 1	Ref		Ref	
	Level 2	0.84 (0.54, 1.31)	0.445*	0.87 (0.55, 1.36)	0.889*
	Level 3	0.45 (0.22, 0.88)	0.024*	0.46 (0.22, 0.91)	0.090*
	Level 4	1.25 (0.69, 2.25)	0.456*	1.29 (0.68, 2.41)	0.804*
Medication adherence^b^ (*N* = 201)			< 0.001		< 0.001
	rcs(PAM-13, 4)	0.27 (0.15, 0.40)		0.25 (0.13, 0.37)	
	rcs(PAM-13, 4)’	− 2.27 (− 3.67, − 0.87)		− 2.21 (− 3.60, − 0.82)	
	rcs(PAM-13, 4)’’	6.77 (2.31, 11.24)		6.69 (2.27, 11.12)	

*rcs* restricted cubic spline, *IRR* incidence rate ratio, *CI* confidence interval, *PAM-13* 13-item Patient Activation Measure

^a^Adjusted for age (centered and as a linear term) and education level

^b^Adjusted for age (centered and as 3-knot rcs terms), education level and persons living with (self, others, family [partner, children, parent])

^c^The number of hospital admissions, emergency department visits, and missed renal appointments were counted over the last 12 months

*Multiple comparison with control adjusted using Dunnett’s contrast

### PAM-13 and treatment adherence

After adjusting for multiple tests, we found that the number of missed appointments in the last 12 months in patient activation level 3 was 46% of those with a patient activation level 1 (95% CI 0.22–0.91), *P* = 0.09 (Table 3), showing no statistically significant association between patient activation level and missed renal appointments in this study.

**Fig. 2:**
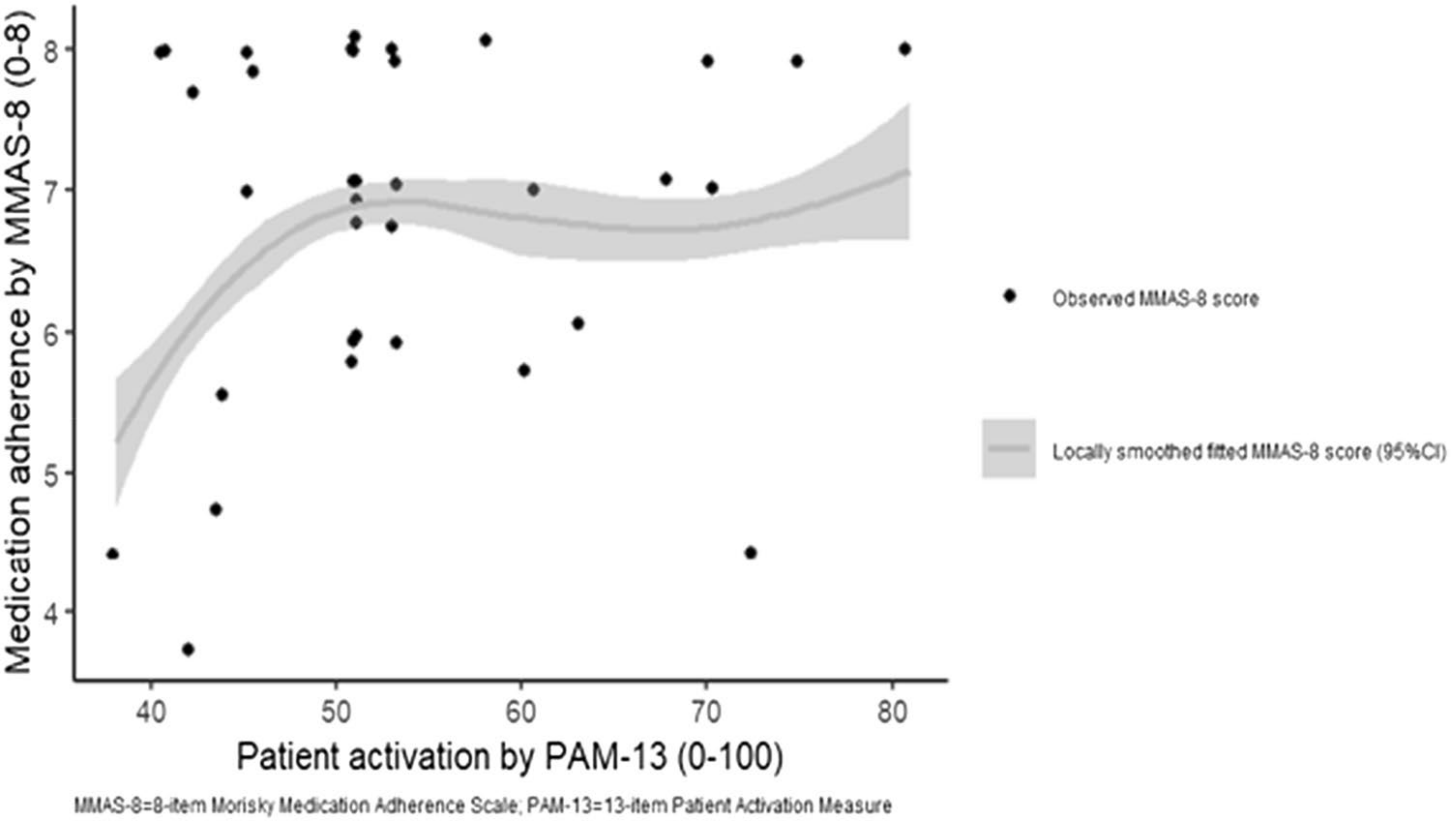
Relationship between pateint activation and medication adherence. In patients who are 65 years or older, with high school education and living with families (*N* = 35)

Patient activation was associated with medication adherence level (*P* < 0.001); however, the relationship was non-linear (Fig. 2). Medication adherence score increased quickly until patient activation score reached 47. Medication adherence score then increased at a slower rate until plateauing at 51. Thereafter, medication adherence remained high.

## Discussion

This study found that most people with stage 5 CKD not on dialysis have low levels of patient activation. Thus, they lack the skills, knowledge, and motivation to take an active role in CKD self-management. Patient age and education were strongly associated with levels of patient activation, as were the number of hospital emergency department visits, and medication adherence. These findings provide an insight into this vulnerable patient cohort imminently approaching kidney failure which often requires complex and demanding kidney treatment options.

Prior studies that investigated patient activation in people with CKD have examined combined CKD stages [9, 11, 26, 28] and dialysis cohorts [13, 18]. However, there have been no studies that considered stage 5 CKD alone. Wilkinson [28] investigated CKD across combined CKD stages 4 and 5 (excluding dialysis) and found that 63% had low levels of activation. This suggests that patient activation may deteriorate as people move into stage 5, given that we found a higher percentage (73%) of low level activation in stage 5 only. Although more people were in the low activation category, mean scores were similar to Zimbudzi (55) [9] and Bos-Touwen (51) [11] who did not stratify CKD stages 3–5.

Focusing on stage 5 CKD is important because this cohort faces major changes in healthcare, often while experiencing a high burden of symptoms and reduced quality of life [4, 29]. Timely education and coaching interventions may contribute to optimal kidney replacement modality choice, reduce unnecessary hospitalisation and reduce catheter incidence at dialysis start [29].

We found that people with stage 5 CKD over the age of 65 reported lower activation levels. This suggests that older adults lack the skills, knowledge, and motivation to engage in self-care practices that could better prepare them for dialysis. This is concerning given older adults will experience the greatest growth in the prevalence of dialysis over the next ten years and will be less likely to start on a home therapy [30]. Efforts to promote patient activation among older adults with stage 5 CKD yet to undergo dialysis may help better prepare this vulnerable population. In addition, self-management approaches for older individuals require greater upfront education and motivation by kidney health professionals. These efforts can be prioritised and enhanced for patients with low activation levels.

A systematic review and meta-analysis that tested the effectiveness of patient activation interventions compared to usual care on health-related behavioural outcomes in adults with CKD found that education alone was not enough for patients to take an active role in their self-management [3]. The findings of this review suggest that patient activation interventions, when tailored and interactive, are effective in improving self-management and self-efficacy. Although this review identified features of an effective intervention, the inherent difficulties in implementing behavioural change strategies within different sociodemographic, economic and cultural contexts, were identified, perhaps, highlighting the need to develop a prototype patient self-management programme co-designed with consumers and clinicians [3].

In our study, people with lower educational levels had lower patient activation levels. Patients’ educational level is an important characteristic to consider when determining whether additional resources are required to promote patient activation to optimise self-management in this pre-dialysis population. Unfortunately, there are no programmes targeting the elderly population with low educational levels and low activation levels who represent most of this study population with CKD stage 5. This pre-dialysis population requires a solid and realistic understanding regarding decision-making about dialysis initiation, symptom burden, frailty, and prognosis.

Patient activation was associated with medication adherence, with those scoring in the lower two quartiles of patient activation reporting lower self-reported medication adherence. Interestingly, we observed a non-linear relationship between medication adherence and patient activation. Medication adherence was linear until a patient activation score of 51 (when patient activation scores change from low activation to high activation), then it plateaued. This is an important finding given that patient outcomes in CKD depend on patients taking their medicines in line with their prescribed regimen to yield the full benefit of the treatment [31]. Non-adherence to medication is costly for the healthcare service, both through waste and increased ill health [31]. High levels of medication adherence have long been identified as a crucial factor in reducing the progression from advanced kidney failure to dialysis start and/or increasing life expectancy in those who choose not to have dialysis [32]. In our study, living with someone was significantly associated with medication adherence and higher patient activation level. Hence, with the support of the multidisciplinary team there is a need to develop targeted interventions using simple tasks so that they can begin to gain confidence and move from being overwhelmed by their illness to greater independence (including their family/carer if required) to improve medication adherence [14].

Healthcare professionals have an essential role in activating and empowering patients. However, the lack of skills to empower patients and staff resources can be activation barriers. We propose that a co-designed model (patient/nurse) be developed that includes education, motivational interviewing and skill development to increase activation levels. This will likely engage individuals to become more active in their roles rather than passive care recipients [4, 33].

This study has both strengths and limitations. The key strength of this study was the response rate (97.1%) and the focus on stage 5 CKD. Limitations included the convenience sampling that may bias results and limit generalisability, and the small sample size that was restricted by the size of the current kidney care programme. In addition, the study focused on sociodemographic characteristics, and thus non-modifiable clinical comorbidities were not recorded. A further limitation was the focus on those who could understand English.

In summary, this study confirms the low level of patient activation among the CKD pre-dialysis population; this is likely to manifest limited capacity for self-management and informed decision-making. Patient activation scores significantly decreased with increased age and increased with higher educational level. Higher patient activation was associated with fewer hospital emergency department visits and increased medication adherence. This study is the platform for further investigating components that increase engagement in positive health-related behaviours for a more active role in self-management. Therefore, integrating patient activation measures into the clinical practice in advanced CKD populations enhances patient and service outcomes.

### Supplementary Information

Below is the link to the electronic supplementary material.

Supplementary file1 (DOCX 20 KB)

## Data Availability

The datasets used and/or analyzed during the current study are available from the corresponding author on reasonable request.
