# Supplementary material for: Patient activation in advanced chronic kidney disease: a cross-sectional study
Source: J Nephrol. 2024 Feb 12;37(2):343–52. doi: 10.1007/s40620-023-01847-x (PMC11043190; doi:10.1007/s40620-023-01847-x)
Supplement: Supplementary file 1 — Supplementary file1 (DOCX 20 KB) [file 40620_2023_1847_MOESM1_ESM.docx]

**Supplementary Table 1. STROBE Statement Checklist**

|  | **Item No** | | **Recommendation** | **Yes/No &**  **Page number** | |
| --- | --- | --- | --- | --- | --- |
| **Title and abstract** | | 1 | (*a*) Indicate the study’s design with a commonly used term in the title or the abstract | | Yes |
|  |  |  | (*b*) Provide in the abstract an informative and balanced summary of what was done and what was found | | Yes |
| **Introduction** | | | | |  |
| Background/rationale | | 2 | Explain the scientific background and rationale for the investigation being reported | | Yes, 1 |
| Objectives | | 3 | State specific objectives, including any prespecified hypotheses | | Yes, 1 |
| **Methods** | | | | |  |
| Study design | | 4 | Present key elements of study design early in the paper | | Yes, 1 |
| Setting | | 5 | Describe the setting, locations, and relevant dates, including periods of recruitment, exposure, follow-up, and data collection | | Yes, 1 |
| Participants | | 6 | (*a*) Give the eligibility criteria, and the sources and methods of selection of participants | | Yes, 1 |
| Variables | | 7 | Clearly define all outcomes, exposures, predictors, potential confounders, and effect modifiers. Give diagnostic criteria, if applicable | | Yes, 2 |
| Data sources/ measurement | | 8 | For each variable of interest, give sources of data and details of methods of assessment (measurement). Describe comparability of assessment methods if there is more than one group | | Yes, 2 |
| Bias | | 9 | Describe any efforts to address potential sources of bias | | Yes, 5 |
| Study size | | 10 | Explain how the study size was arrived at | | Yes, 3 |
| Quantitative variables | | 11 | Explain how quantitative variables were handled in the analyses. If applicable, describe which groupings were chosen and why | | Yes, 2 & 3 |
| Statistical methods | | 12 | (*a*) Describe all statistical methods, including those used to control for confounding | | Yes, 2 & 3 |
|  |  |  | (*b*) Describe any methods used to examine subgroups and interactions | | Yes, 2 & 3 |
|  |  |  | (*c*) Explain how missing data were addressed | | Yes, 2 & 3 |
|  |  |  | (*d*) If applicable, describe analytical methods taking account of sampling strategy | | Yes, 2 & 3 |
|  |  |  | (*e*) Describe any sensitivity analyses | | N/A* |
| **Results** | | | | |  |
| Participants | | 13 | (a) Report numbers of individuals at each stage of study—eg numbers potentially eligible, examined for eligibility, confirmed eligible, included in the study, completing follow-up, and analysed | | Yes, 3 |
|  |  |  | (b) Give reasons for non-participation at each stage | | Yes, 3 and Figure 1 |
|  |  |  | (c) Consider use of a flow diagram | | Yes, Figure 1 |
| Descriptive data | | 14 | (a) Give characteristics of study participants (eg demographic, clinical, social) and information on exposures and potential confounders | | Yes, 3 &  table 1 |
|  |  |  | (b) Indicate number of participants with missing data for each variable of interest | | Yes, tables |
| Outcome data | | 15 | Report numbers of outcome events or summary measures | | Yes, 3 and tables |
| Main results | | 16 | (*a*) Give unadjusted estimates and, if applicable, confounder-adjusted estimates and their precision (eg, 95% confidence interval). Make clear which confounders were adjusted for and why they were included | | Yes, 2 & 3 |
|  |  |  | (*b*) Report category boundaries when continuous variables were categorized | | Yes, 2 |
|  |  |  | (*c*) If relevant, consider translating estimates of relative risk into absolute risk for a meaningful time period | | Yes, 2 & 3 |
| Other analyses | | 17 | Report other analyses done—eg analyses of subgroups and interactions, and sensitivity analyses | | Yes, page 2 & 3 |
| **Discussion** | | | | |  |
| Key results | | 18 | Summarise key results with reference to study objectives | | Yes, 4 |
| Limitations | | 19 | Discuss limitations of the study, taking into account sources of potential bias or imprecision. Discuss both direction and magnitude of any potential bias | | Yes, 5 |
| Interpretation | | 20 | Give a cautious overall interpretation of results considering objectives, limitations, multiplicity of analyses, results from similar studies, and other relevant evidence | | Yes, 4 & 5 |
| Generalisability | | 21 | Discuss the generalisability (external validity) of the study results | | Yes, 5 |
| **Other information** | | | | |  |
| Funding | | 22 | Give the source of funding and the role of the funders for the present study and, if applicable, for the original study on which the present article is based | | This study had funding secured by the RAH Research Committee - 2022 RRC Allied Health, Pharmacy and Nursing Clinical Research Grant. MYIP: 15816 |

*N/A=not applicable
